# Notes upon a Characteristic

**Published:** 1887-07

**Authors:** 


### NOTES UPON A CHARACTERISTIC.

The symptom of Lycopodium, "fan-like motion of alæ nasi," has justly been considered a valuable characteristic. Other remedies have this symptom but none so characteristically as Lyc. In our May issue, Dr. Sherbino mentions Bapt., Lyc., and Phos. Ant-tart. and Bromine also have it. Dr. E. A. Ballard wrote, mentioning Ammoniacum and Chelidonium, and gave Hering's *Guiding Symptoms* as his authority.

Later, Dr. A. F. Randall writes he has also noted Chel. as having this symptom. Hering gives this symptom under Ant-tart. as a concomitant of whooping-cough, and also in pneumonia, in which the nostrils "inflated and moved rapidly, like wings."

